# MEALTIME BEHAVIORS AND FOOD INTAKE IN OLDER ADULTS WITH DEMENTIA: IMPACT OF PERSON-CENTERED AND TASK-CENTERED CARE

**DOI:** 10.1093/geroni/igae098.1098

**Published:** 2024-12-31

**Authors:** Wen Liu, Kyuri Lee, Heather Suh

**Affiliations:** University of Iowa, Iowa City, Iowa, United States; University of Iowa, Iowa City, Iowa, United States; University of Iowa, Iowa City, Iowa, United States

## Abstract

Nursing home residents with dementia experience mealtime behaviors and low food intake due to multilevel factors, among which mealtime caregiving is critical. Prior work supported associations between staff mealtime care and resident mealtime behaviors and food intake using small samples of meal-segments. This study examined their associations using 197 full-meal videos (duration=37.85±18.24 minutes), capturing 47 staff (age 45.6±11.8 years; 92.3% female; 80.8% white; 15.5±12.9 caregiving years) and 12 residents with moderately-severe to severe dementia (age 71.9±7.5 years; 91.7% male; 91.7% white). Staff mealtime care approaches (n=12,800) were mostly person-centered addressing modifications of resident abilities (70.3%), dyadic interactions (12.1%), and dining environment (6.8%), followed by task-centered care (TCC, 10.8%). Resident mealtime behaviors (n= 9,424) were resistive behaviors (21%), functional impairments (13.2%), chewing/swallowing difficulties (48.1%), and positive behaviors (17.6%). Resident food intake was 626.23±353.17 gram/meal. Resistive behaviors were associated with fewer modifications of resident abilities (b=-.215, p=.053) and more TCC (b=.202, p=.058). Functional impairments were associated with more modifications of resident abilities (b=.186, p=.048) and more TCC (b=.446, p<.001). Chewing/swallowing difficulties were associated with more modifications of resident abilities (b=.287, p=.006). Positive behaviors were associated with fewer modifications of resident abilities (b=-.310, p=.002) and more modifications of dining environment (b=.330, p=.001). Food intake was associated with more modifications of resident abilities (b=.289, p=.009). Minimizing use of task-centered care may help improve mealtime challenging behaviors. While modifications of resident abilities may not address functional impairments, they help manage resistive behaviors and improve food intake. Modifications of dining environment may help promote positive behaviors.

